# The Emerging Role of Vitamin D and Vitamin D Receptor in Diabetic Nephropathy

**DOI:** 10.1155/2020/4137268

**Published:** 2020-07-11

**Authors:** Min Lei, Zhangsuo Liu, Jia Guo

**Affiliations:** ^1^Department of Nephrology, The First Affiliated Hospital of Zhengzhou University, 1 East Jianshe Road, Zhengzhou, Henan 450052, China; ^2^Key Laboratory of Precision Diagnosis and Treatment for Chronic Kidney Disease in Henan Province, Zhengzhou 450052, China; ^3^Research Center for Kidney Disease, Henan Province, Zhengzhou 450052, China

## Abstract

Diabetic nephropathy (DN), one of the most common and severe microvascular complications of diabetes mellitus (DM), is an important risk factor for DM patient's death. Nowadays, DN has become the leading cause of end-stage renal disease (ESRD) in most countries without effective therapeutic methods. Recently, the renoprotective effects mediated by vitamin D (VD) and vitamin D receptor (VDR) have been evidenced. VD, a kind of steroid with the active form 1,25(OH)_2_D_3_, has been known for the crucial roles in the modulation of serum calcium and phosphorus concentrations. It exerts important functions by binding with its receptor VDR.VDR, a transcription factor located at chromosome 12 containing 9 exons, is one of the nonsteroid nuclear hormone receptor superfamily, which participates in transcriptional regulation of genes in tissue- and cell-specific ways. Increasing evidences have demonstrated that VD/VDR signaling pathway possesses a variety of kidney-protective effects in DN patients, such as antiproteinuria, antifibrosis, anti-inflammatory, and preventing podocyte damage. Although there are many studies on the role of the VD/VDR signaling pathway in DN, the effects and mechanisms still need to be further explained. This review summarized the multiple roles of VD/VDR in podocyte injury, tubule lesions, interstitial fibrosis, and inflammation, as well as the clinical applications about DN to explore much more and effective therapeutic methods for DN.

## 1. Introduction

Diabetes mellitus (DM) is an endocrine system disease, and its incidence is increasing year by year, which is the eighth leading cause of death in the world [1]. According to reports in the literatures, the number of people with diabetes worldwide was about 382 million in 2013. In 2012 and 2013, diabetes caused 1.5-5.1 million deaths per year, and in 2014, it caused 422 million deaths per year. It is estimated that 592 million will die of diabetes in 2035 [2]. Diabetic nephropathy (DN), the leading cause of end-stage renal disease (ESRD), has become one of the most common and severe microvascular complications of diabetes [3–5]. Therefore, early diagnosis and treatment can improve patients' quality of life and survival. Although some treatments could delay the onset and progression of DN, the incidence of DN is still increasing every year, causing serious consequences and economic burden. A growing number of clinical and basic studies have shown that vitamin D (VD) and its analogs have impressive kidney-protective effects, such as antiproteinuria, antifibrosis, anti-inflammatory, and preventing podocyte damage by binding to VDR. But the mechanisms of VD and VDR in different pathological processes in DN are still unclear. This review summarizes the currently important and novel research on the roles, mechanisms, and clinical applications of VD/VDR signaling pathway in DN, in order to further study the multiple and crucial roles of VD and VDR.

## 2. Vitamin D and Vitamin D Receptor

VD is one kind of steroid hormone with active form 1,25(OH)_2_D_3_ which is also called calcitriol [6–8]. VD has been known for its important role in regulating blood calcium and blood phosphorus concentrations [9]. Human beings can produce VD from 7-dehydrocholesterol in the skin by the effect of ultraviolet rays (10 nm-400 nm). Little VD can be absorbed from foods such as fatty fish. VD is converted to 25-hydroxyvitamin D (25OH-D) by the influence of 25-hydroxylase in the liver, subsequently turned into the active form 1,25(OH)_2_D_3_ by 1*α*-hydroxylase (mainly CYP27B1) in human renal proximal tubular epithelial cells (PTECs) [6, 10]. The biological effects of VD are realized by combining with VDR, a transcription factor located at chromosome 12 containing 9 exons. It is expressed in a variety of tissues and cells, such as intestines, bones, parathyroid glands, kidneys [3, 11], reproductive system [6, 12], and immune cells (e.g., B lymphocytes, T lymphocytes, and antigen-presenting cells) [13, 14]. As one of the nonsteroid nuclear hormone receptor superfamily, VDR is found to bind with target DNA sequences and acts as a heterodimer with the retinoid X receptor (RXR), recruiting cofactors to form a transcriptional complex and inducing target gene expression [6, 12, 15–17]. The multiple effects of VD/VDR include maintaining mineral homeostasis and regulating inflammatory, immune responses and may improve response to cancer [10, 11, 17]. However, VDR participates in transcriptional regulation of genes in tissue- and cell-specific ways [6, 11, 15–17]. According to many publications, VD/VDR is inextricably linked to the development of many diseases. Lots of studies have showed that VD/VDR plays a crucial role in the physiological and pathological processes of cancer [8, 16, 18–21], DN [3, 22, 23], autoimmune diseases, inflammation, infection, cardiovascular disease [1, 24], hereditary 1,25-dihydroxyvitamin D-resistant rickets (HVDRR) [2], etc. O. Teriolo et al. have reported the role of VD/VDR in gynecological diseases, such as breast cancer, ovarian cancer, polycystic ovary syndrome, and endometriosis. Some researchers have proposed that paricalcitol, the VD analog, plays an anti-inflammatory role which may depend on the inhibitory effect of transforming growth factor-*α*/ADAM17/the epidermal growth factor receptor (TGF-*α*/ADAM17/EGFR) pathways stimulated by aldosterone. G. Ferlazzo et al. have suggested the role of VD deficiency in the development of inflammatory bowel disease (IBD) with regard to immune regulation. In addition, some studies support the relationship between VD and cardiovascular diseases such as left ventricular hypertrophy, atrial fibrillation, the metabolic syndrome, and peripheral artery disease [25]. In the kidney, VDR has been shown to be expressed in the proximal and distal tubular epithelial cells, in the glomerular parietal epithelium, in collecting duct cells, and also in mesangial cells and podocytes, as well as juxtaglomerular apparatus [26].

## 3. Diabetic Nephropathy

DN is considered to be a complex disease. It is a multifactorial disease caused by the interaction of genetic and environmental factors and is characterized by progressive albuminuria and a gradual decline in glomerular filtration rate (GFR) [4, 26]. From the perspective of pathology, the occurrence of DN is due to glomerular and tubular hypertrophy, the glomerular capillary basement membrane thickening, mesangial matrix hyperplasia, podocyte injury, endothelial dysfunction, macrophage infiltration, renal tubulointerstitial fibrosis, and glomerular sclerosis. Progressive albuminuria is generally considered the main and initial clinical manifestation [3, 27].

There is a great diversity of pathological processes leading to DN. First of all, it is the hemodynamic pathway referring to the activation of the local renin-angiotensin-aldosterone system (RAAS) in podocytes that can lead to DN. The formation of angiotensin II in the kidney not only controls glomerular hemodynamics and renal tubular sodium transport but also activates many inflammation and fibrotic pathways [28]. On the other side, hyperglycemia stimulates PTECs and mesangial cells [29] to produce angiotensin II (AngII) and activate the renin-angiotensin-aldosterone and urotensin systems. Secondly, various protein kinase pathways can also promote the development of DN such as protein kinase C (PKC), the Janus kinase/signal transducers/activators of transcription (JAK-STAT), nuclear factor kappa light-chain enhancer of activated B cells (NF-*κ*B), and p38 mitogen-activated protein kinase pathways as well as intracellular activated polyol pathways [30]. Thirdly, the advanced glycation end products (AGEs) and oxidative stress by reactive oxygen species (ROS) are another causes of DN. Serum advanced oxidizing protein products (AOPP) are new markers of protein damage induced by oxidative stress. They are similar to AGEs structurally and show biological activity. Studies have shown that the long-term accumulation of AOPPs in plasma may promote renal inflammation in patients with DM by activating renal nicotinamide adenine dinucleotide phosphate (NADPH) oxidase. Rong et al. proved that endoplasmic reticulum stress and podocyte apoptosis secondary to the accumulation of AOPPs lead to the worsening of DN. Conti et al. have found that oxidative stress was higher in patients with DM than in healthy people; especially, it appeared to be more severe in patients with renal complications [30]. What is more, there are inflammatory pathways in DN including the effects of transforming growth factor-*β* (TGF-*β*), TNF-*α*, IL-1, TLR4, adiponectin, and nuclear hormone receptors [26, 27]. ROS is another important pathway leading to DN. Hyperglycemia can cause mitochondrial overload and then ROS production, which in turn damages glomerular podocytes and promotes their apoptosis [31].

## 4. The Role of VD/VDR Signaling Pathway in Diabetic Nephropathy

Recently, increasing studies have demonstrated the protective effect of active VD and its analogs on renal functions. Strong antiproteinuria activity and prevention of podocyte injury were found in experimental animal models of renal disease, including unilateral ureteral obstruction models, 5/6 nephrectomized rats, adriamycin-induced nephropathy model, and puromycin aminonucleoside-induced apoptosis in podocytes [32].

The Third National Health and Nutrition Examination Survey (NHANES III) found that decreasing 25(OH)D concentration is associated with an increasing prevalence of albumin urination in the general population. Assessments of people with diabetes showed an independent association between VD deficiency and DN. With the DN progressing, the insufficiency of VD is more serious [26]. The prevalence of VD deficiency is very high in patients with kidney disease. It is because reduced CYP27b1 activity in human renal PTECs inhibits the production of 1,25(OH)_2_D_3_ while impairing the function of reabsorption of 25(OH)D. Meanwhile, the elevated levels of fibroblast growth factor inhibit the biosynthesis of 1,25(OH)_2_D_3_ [33].

Clinical studies showed that the survival rates of chronic kidney disease (CKD) patients could be enhanced by active VD treatment, because VD deficiency is one of the most important risk factors for patients with CKD [34]. VD and its analogs considerably improve renal function by reducing urinary albumin creatinine ratio (UACR) and improving estimated glomerular filtration rate (eGFR) in patients with CKD [26]. The mechanisms are dependent not only on the regulation of calcium, phosphorus, and parathyroid hormone (PTH) levels in the blood but also on the reduction of glomerular sclerosis and interstitial fibrosis by 1,25(OH)_2_D_3_ [34]. In DN animal models, albumin excretion rate, glomerular base membrane thickness, and total kidney volume that were pretty reduced by 1,25(OH)_2_D_3_ treatment [33].

In order to better assess the effect of the VD/VDR signaling pathway in the kidney, after overexpression of VDR in podocyte-specific knockout VDR (VDR-KO) mice, the albuminuria was significantly reduced, accompanied with some improved symptoms compared with VDR-KO mice, such as the disappearance of podocyte foot processes, the glomerular basement membrane (GBM) thickening, glomerular sclerosis, an increase in glomerular fibronectin levels, and a decrease of nephrin expression. These phenomena suggest the significant effects of VD/VDR expressions in maintaining renal health [35].

## 5. The Mechanism of VD/VDR in Diabetic Nephropathy

### 5.1. The Mechanism of VD/VDR in Podocyte Injury

In the causes of kidney damage, podocytes serve as the last gate of the glomerular filtration barrier, and their damage will lead to occurrence of proteinuria, which will further increase kidney damage. DN is considered one kind of podocyte disease [3]. Podocytes, the outermost layer of glomerular filtration, are highly differentiated epithelial cells in the glomerular basement membrane playing a key role in the regulation of glomerular filtration in the kidney. The foot processes are a vital part of the glomerular filtering barrier, preventing proteins and other macromolecules from being filtered into the urine. According to some reports, 1,25(OH)_2_D_3_ could promote the expression of nephrin which are key components of the slit diaphragm formed between adjacent crossed foot processes. As the main size and charge-selective barrier of proteins, podocyte injury will lead to proteinuria and worsen DN and eventually develop into ESRD [36]. Reducing urinary albumin excretion through any intervention leads to a reduced incidence of these outcomes [37].

In recent years, there is growing evidence showed that podocytes express VDR and the VD/VDR signaling pathway has potent renal protective activity against DN. There are studies that have reported that paricalcitol, the activator of VDR (VDRA), significantly ameliorated urinary albumin excretion of 10-week-old db/db mice after only four-week treatment [3]. Another report had showed that VDR could directly bind with the acetylheparinase promoter and inhibit the activity of the acetylheparinase gene promoter in podocytes, leading to the decrease of proteinuria in DN [33]. Furthermore, some researches indicated that after the treatment of VDRA, the expression of nephrin was enhanced, accompanied with the improvement of podocyte injury and proteinuria in the isolated glomerulus of DN rats, with the activation of the PI3K/AKT signaling pathway as the underlying mechanism [38].

On the one hand, the antiproteinuria effect of VD and its analogs is due to inhibition of hyperglycemia-induced podocyte apoptosis [35]. Researchers found that 1,25(OH)_2_D_3_ suppresses high glucose-induced podocyte apoptosis by abrogating ERK phosphorylation, inhibiting p38 activity [36], and targeting the activation of the NF-*κ*B pathway [33]. The Bcl2 family members play important roles in apoptosis. 1,25(OH)_2_D_3_ could alleviate hyperglycemia-induced podocyte apoptosis by increasing the levels of Bcl2 (antiapoptotic) and decreasing the levels of Bad and Bak (proapoptotic) [39]. It is universally acknowledged that the RAAS is another cause of promoting podocyte apoptosis and thus mediates the injury of DN. The expression of renin and angiotensinogen can be induced by high glucose (HG) in podocytes cultured in vitro. It has been proved that 1,25(OH)_2_D_3_ can block increased intracellular renin activity, the levels of AngII released extracellularly under HG exposure, and the expression of AT1 receptors [36]. The interaction between plasma renin and VD is closely related to the state of VDR. When VD is deficient, both unliganded and ligand VDR decrease, and the lack of ligand VDR improves the transcription of renin, while unliganded VDR can enhance the transcription of angiotensinogen and angiotensin II type I receptors (AT1Rs) by regulating the expression of p53. Both pathways lead to activation of RAAS, and VD could become a regulator of RAAS by controlling VDR. A variety of studies have shown that reducing urinary albumin excretion by inhibiting RAAS is associated with preservation of renal function. There is a better reduction in albuminuria when RAAS inhibitors are combined with VD in DN [40]. The VDR-KO mice were more susceptible to hyperglycemia-induced kidney damage [41, 42]. Due to glomerular basement membrane thickening and podocyte injury, VDR-KO diabetic mice develop more severe albuminuria and glomerular sclerosis and increased expression of fibronectin (FN) than diabetic wild-type mice [41]. Meanwhile, the local renal RAAS activation could increase the expression of TGF-*β*, angiotensinogen, renin, and connective tissue growth factor, accompanied with more severe kidney damage. It would lead to early-onset albuminuria, interstitial fibrosis, and glomerular sclerosis [26]. In short, these data indicated that the VD/VDR signal pathway could attenuate HG-induced podocyte apoptosis by inhibiting the RAAS system.

On the other hand, VD and its analogs could reduce proteinuria by attenuating podocyte injury. It is thought that ion channel dysfunction may also be one of the mechanisms of podocyte injury. Some studies demonstrated that the elevated expression of TRPC6 (transient receptor potential cation channel, subfamily C, member 6) plays an important role in the pathogenesis of progressive podocyte injury in DN. The expression of TRPC6 was positively correlated with 24-hour proteinuria and desmin, a marker protein of podocyte injury, in streptozotocin- (STZ-) induced DN rats. In contrast, it was negatively correlated with VDR and nephrin expression. Treatment with calcitriol can attenuate podocyte injury by inhibiting the enhanced expression of TRPC6 in DN [32]. Present studies have proved that calcitriol and paricalcitol could reactivate and maintain the expressions of podocyte-specific components, preventing selective permeability of the renal barrier. Meanwhile, other studies showed that calcitriol could normalize the expression of podocyte marker protein including nephrin and podocin in DN rats accompanied with the reduction of proteinuria [43].

In order to verify whether the increase of VDR expression in podocytes could enhance the renoprotective effect of VD, some researchers compared the therapeutic effectiveness of low-dose VD analogs doxercalciferol (Dox) in STZ-induced wild-type (WT) diabetes and VDR-overexpression mice. The results showed that low-dose Dox had little effect on the development of albumin urination in WT mice, but it almost completely blocked albumin urination in VDR-overexpression mice. Therefore, overexpression of VDR in podocytes result in more sensitive to VD therapy in mice.

Emerging evidence indicates that the podocyte epithelial-to-mesenchymal transition (EMT) has emerged as a potential pathway leading to proteinuria in DN [44], because of podocyte dysfunction caused by the loss of the podocyte-specific markers nephrin and podocin [44, 45]. The activation of Wnt/*β*-catenin pathway was considered one of the key factors in podocyte EMT process. As a crucial protein in the Wnt/*β*-catenin pathway, glycogen synthase kinase 3 beta (GSK-3*β*) plays important roles in podocyte injury. Researchers tried to use different ways to inhibit the activation or expression of GSK-3*β* in order to observe the podocytes' protective effects. Recent studies have revealed that (2′Z,3′E)-6-bromoindirubin-3′-oxime (BIO), an inhibitor of GSK-3*β*, was able to inhibit HG-induced EMT in podocytes and the renal cortex. The treatment of BIO protects kidney function by maintaining integrity of filtration membrane and reducing urinary albumin excretion rate (UAER), but not influencing blood glucose level [32]. Guo et al. have recently reported that in the Wnt/GSK-3*β*/*β*-catenin signaling pathway, with the downregulation of GSK-3*β* expression, VDR expression was increased, leading to its renoprotective effect [3].

### 5.2. The Mechanism of VD/VDR in Renal Tubule Lesions

So far, there were only a few reports on the mechanism of the VD/VDR signaling pathway in renal tubules in DN. The active form of VD is produced by mitochondria of the renal proximal convoluted tubules. VD plays a pivotal role in renal tubules, and VDR is present in both proximal and distal nephron. Megalin-cubilin-amnionless is a kind of VD-related mechanism that can regulate and ameliorate the renal tubular injury response. Most VD moves in the circulation by combining with VD-binding protein (DBP). VD-DBP complex is constantly filtered through the glomerulus and endocytosed via megalin into PTECs, leading to activation of 25(OH)D_3_ into the active form [46]. Recently, some studies have indicated that the haptoglobin (Hp) genotype (1-1 and 2-2) is the main determinant of DN progress which can bind with hemoglobin (Hb). It can prevent heme-iron-mediated oxidation accompanied by tissue damage. As expected, the Hb clearance function of diabetic mice with genotype HP 2-2 is impaired. For instance, the increase in iron deposits and oxidative stress in the proximal convoluted tubules (PCT) leads to a decrease expression of *α*-klotho and VDR in renal tubules, accompanied with increasing PCT damage and worsening kidney injury, thereby increasing the incidence of DN and its complications among Hp 2-2 genotype patients [22]. Besides, other studies have demonstrated that microtubule-associated protein 1 light chain 3 (LC3), which acts as a VDR-binding protein for transporting to the nucleus, could inhibit HG-induced fibrotic gene expressions in HK-2 cells by promoting the VDR-RXR dimer formation [47]. In addition, some researchers have put forward that active VD and VDR help preventing high glucose-induced oxidative stress and apoptosis in human tubular epithelium cells via upregulating the protein kinase B/mitochondrial uncoupling protein 2 (AKT/UCP2) signaling pathway [48]. As a target of ROS, AKT plays a critical role in controlling cell growth and proliferation, and its downregulation contributes to the apoptosis of proximal tubular cells in the kidney. UCP2, a kind of protein, mainly located in proximal convoluted tubule cells, plays a key role in the preservation of mitochondria, immune regulation, and modulation of oxidative stress affecting the kidney physiologically or pathologically. 1,25(OH)_2_D_3_ exerts a direct protective effect, dramatically decreasing the levels of IL-6 and IL-8 in PTECs and modulating the activity of effector T-cells [46]. These studies suggested that VD/VDR also played an important protective role in the renal tubules of DN.

### 5.3. The Mechanism of VD/VDR in Interstitial Fibrosis

Studies have found that the expression of VDR in renal tissue was significantly decreased in the mice suffering from renal interstitial fibrosis, which indicated the importance of VDR. The downregulation of VDR could be rescued by treating one kind of active vitamin analog paricalcitol. In addition, the therapeutic effect of paricalcitol on renal interstitial fibrosis has also been significantly associated with its regulation effect on VDR expression. There are many reports demonstrating that 1,25(OH)_2_D_3_ combined with VDR could inhibit the progression of DN fibrosis by inhibiting the production of FN and the activation of TGF-*β* and RAAS in high glucose-treated mesangial cells [41]. It can also directly block TGF-*β*-induced EMT and extracellular matrix (ECM) proteins in cultured tubular cell via antagonizing NF-*κ*B activity. What is more, 1,25(OH)_2_D_3_ can improve renal fibrosis via reducing the expression of collagen, other key profibrotic factors, and increasing the expression of antifibrotic factors, such as BMP7 and MMP8. VDR also directly inhibits the expression of Snail and stimulates E-cadherin expression in primary tubular cell cultures as a means of EMT prevention. In conclusion, these researches affirmed the protective role of VDR in inhibiting renal fibrosis [42, 49].

### 5.4. The Mechanism of VD/VDR in Anti-Inflammation

Nowadays, inflammation is considered to be the main pathological cause of the onset and progression of DN and is also involved in the all-cause poor prognosis of diabetes [37]. Inflammation is a universal initial response that is triggered by harmful stimuli and conditions [50]. Increased inflammation is regarded as a hallmark of diabetes and plays a key role in the development and progression of DN. Expression of proinflammatory cytokines, chemokines, and cell adhesion molecules is increased in the serum and urine of patients with diabetes and DN. Reducing inflammatory processes can either alleviate or prevent the development of kidney damage in animal models of DN [51].

Recent studies have shown that VDR agonists can effectively alleviate the progression of DN by reducing renal inflammation in DN [35]. Inflammation is involved in the pathogenesis of DN through various proinflammatory cytokines, including interleukin-1*β* (IL-1*β*), interleukin-6 (IL-6), interleukin-18 (IL-18), monocyte chemoattractant protein-1 (MCP-1), and tumor necrosis factor-*α* (TNF-*α*). Moreover, transcription factor NF-*κ*B is an important inflammatory stimulus for DN, which regulates adhesion molecule gene expression chemokines and cytokines [52]. Emerging evidences have showed that VDR activation plays an anti-inflammatory role by inhibiting the activation of NF-*κ*B in tubular and mesangial cells [33, 34]. VDR also plays an anti-inflammatory role by regulating the function of antigen-presenting cells, interfering the maturation of dendritic cells, and inhibiting the expression of interleukin 12 (IL-12) as well as transcription genes that encode leukocyte interleukin 2 (IL-2) and interferon *γ* (INF-*γ*).

Researchers have proved that VD agonists reduced the expression of inflammatory mediators' fibronectin and renin in cultured podocytes and decreased glomerular inflammation in a rat experimental model of diabetes induced by STZ. Paricalcitol and calcitriol could prevent the increase in MCP-1, IL-6, renin, and fibronectin mRNA expression and the secretion of MCP-1 to the culture media induced by high glucose in cultured podocytes. In brief, VDR activation has local renal anti-inflammatory effects in DN rats [34].

## 6. The Role of VD/VDR of the Clinical Treatment in Diabetic Nephropathy

VD is widely recognized as renal protective. However, whether VD supplementation provides benefits to DN patients remains controversial. Researchers conducted a meta-analysis to systematically assess the effects of VD supplements on renal function, inflammation, and glycemic control in DN patients. The results showed that VD supplements including calcitriol and alfacalcidol and 1,25(OH)_2_D_3_ are beneficial for 24-hour urine protein and inflammation index and lessen high-sensitivity C-reactive protein (hs-CRP) but have no effects on hemoglobin A1c (HbA1c), serum creatinine (SCr), eGFR, and glycemic control index in DN patients. Besides, analysis based on different types of VD supplementations showed significant decreases of UAER and TNF-*α* in patients assigned to receive calcitriol [53].

Paricalcitol, a third-generation VD analog, is wildly used to prevent and treat DN leading to fewer episodes of hyperphosphatemia and hypercalcemia compared with calcitriol [54]. According to the above review, we have mentioned that paricalcitol has pleiotropic and antioxidant effects on cellular homeostasis. Studies have proved that paricalcitol improves glomerular damage and tubular toxicity in DN rats induced by STZ [55]. However, the role of VD and its analog in the clinic is not clear. In 2010, a large-scale randomized controlled study (VITAL) showed that both type 2 diabetes and DN patients treated with 2 *μ*g paricalcitol for 24 weeks got a reduction of residual albuminuria excretion. Across the entire treatment phase, the UACR has also been decreased significantly and reached its peak of -28% in the 12th week. In addition, eGFR and systolic blood pressure decreased substantially at the 4th week and then remained stable. After drug withdrawal, it is worth noting that all measurements including albuminuria excretion, UACR, eGFR, and systolic blood pressure returned towards baseline suggesting that the effects of paricalcitol were real and reversible [56]. In another double-blind randomized study of 281 patients with type 2 diabetes already receiving angiotensin-converting enzyme inhibitor (ACEI) and/or angiotensin receptor blocker (ARB) therapy, the use of paricalcitol reduced albuminuria significantly. It was the first clinical test of the hypothesis that underlying paricalcitol possesses pleiotropic effects and can influence albuminuria in the case of a stable dose of ACEI and/or ARB treatment [57]. There is a study suggesting that high-dose supplementations of VD is associated with albuminuria reduction and the amelioration of renal injury in type 1 diabetes mellitus (T1DM) [58]. Subsequently, other researchers studied the effects of VD on bone mineral density (BMD) and bone metabolism in patients with type 2 diabetic nephropathy (T2DN). The results showed that VD treatment could increase bone mineral density and improve bone metabolism in patients with T2DN, meanwhile leading to the decreased levels of osteoprotegerin (OPG), bone gla protein (BGP), intact parathyroid hormone (iPTH), C-terminal telopeptides of type I collagen (*β*-CTX), pyridinoline (Pyr)/Cr, and deoxypyridinoline (D-Pyr)/Cr. However, the level of procollagen type I N-propeptide (PINP) was increased. This effect was more pronounced in the treatment of VD combined with pioglitazone hydrochloride (PIO). PIO is defined as a peroxisome proliferator-activated receptor-*γ* (PPAR-*γ*) agonist that is involved in glucose metabolism by ameliorating insulin sensitivity. It has shown a renal protective effect. It suggests that VD and PIO combination therapy for T2DN is more effective in improving BMD and bone metabolism than using VD or PIO, respectively, in treating T2DN patients [59].

## 7. Conclusion

In recent years, CKD has become a global public health problem, and the incidence of ESRD is increasing. DN is the main cause of ESRD so it is extraordinarily significant to study the new effective treatment of DN.

It is now well recognized that VD/VDR plays an important role not only in regulating blood calcium and phosphorus levels but also in the progression of many other diseases such as kidney diseases, especially in DN. Whether from the perspective of basic research or clinical research, the use of VD and VDR activator can improve the clinical symptoms and consequences of DN by antiproteinuria, antifibrosis, anti-inflammatory, preventing podocytes damage, and so on. In summary, we consider that VD and VDR have shown an increasingly important protective role in DN through multiple mechanisms of action.

## Abbreviations

ACEI: Angiotensin-converting enzyme inhibitor
AGEs: Advanced glycation end products
AKT/UCP2: The protein kinase B/mitochondrial uncoupling protein 2
AngII: Angiotensin II
ARB: Angiotensin receptor blocker
AT1Rs: Angiotensin II type I receptors
*β*-CTX: C-terminal telopeptides of type I collagen
BGP: Bone gla protein
BIO: (2′Z,3′E)-6-bromoindirubin-3′-oxime
BMD: Bone mineral density
CKD: Chronic kidney disease
DBP: VD-binding protein
DKA: Diabetic ketoacidosis
DM: Diabetes mellitus
DN: Diabetic nephropathy
Dox: Doxercalciferol
D-Pyr: Deoxypyridinoline
ECM: Extracellular matrix
eGFR: Estimated glomerular filtration rate
EGFR: The epidermal growth factor receptor
EMT: Epithelial-to-mesenchymal transition
ESRD: End-stage renal disease
FN: Fibronectin
GBM: Glomerular basement membrane
GFR: Glomerular filtration rate
GSK-3*β*: Glycogen synthase kinase-3 beta
Hb: Hemoglobin
HbA1c: Hemoglobin A1c
HG: High glucose
Hp: Haptoglobin
hs-CRP: High-sensitivity C-reactive protein
HVDRR: Hereditary 1,25-dihydroxyvitamin D-resistant rickets
IBD: Inflammatory bowel disease
IL: Interleukin
INF-*γ*: Interferon *γ*
iPTH: Intact parathyroid hormone
JAK-STAT: Janus kinase/signal transducers/activators of transcription
LC3: Light chain 3
MCP-1: Monocyte chemoattractant protein-1
NADPH: Nicotinamide adenine dinucleotide phosphate
NF-*κ*B: Nuclear factor kappa light-chain enhancer of activated B cells
NHANES III: The Third National Health and Nutrition Examination Survey
OPG: Osteoprotegerin
PCT: Proximal convoluted tubules
PIO: Pioglitazone hydrochloride
PPAR-*γ*: Peroxisome proliferator-activated receptor-*γ*
PTECs: Proximal tubule epithelial cells
PTH: Parathyroid hormone
Pyr: Pyridinoline
RAAS: Renin-angiotensin-aldosterone system
ROS: Reactive oxygen species
SCr: Serum creatinine
T1DM: Type 1 diabetes mellitus
T2DN: Type 2 diabetic nephropathy
TGF-*α*: Transforming growth factor-*α*
TGF-*β*: Transforming growth factor-*β*
TNF-*α*: Tumor necrosis factor-*α*
UACR: Urinary albumin creatinine ratio
UAER: Urinary albumin excretion rate
VD: Vitamin D
VDR: Vitamin D receptor
VDRA: Vitamin D receptor activator
VDR-KO: VDR knockout
WT: Wild type.
